# Positive Emodiversity Buffers the Association Between Stress and Depressive Symptoms

**DOI:** 10.1093/geroni/igaa057.2250

**Published:** 2020-12-16

**Authors:** Lizbeth Benson, Anthony Ong

**Affiliations:** 1 The Pennsylvania State University, University Park, Pennsylvania, United States; 2 Cornell University, Ithaca, New York, United States

## Abstract

Intensive measurements of individuals’ experiences allow for identifying patterns of functioning that may be markers of resilience, and whether such patterns differ across the life span. Using 8 daily diary reports collected in the second burst of the National Study of Daily Experiences (NSDE, n=848, age 34-84; 55%female), we examined whether positive emodiversity (Shannon’s entropy) attenuated the association between cumulative stressor exposure and depressive symptoms, and age-related differences therein. Results indicated age moderated the extent to which positive emodiversity attenuated the association between stress and depressive symptoms (b=0.11, p < .05). The attenuated association was strongest for younger adults with higher positive emodiversity, compared to those with lower positive emodiversity. For older adults, the association between stress and depressive symptoms was relatively similar regardless of their positive emodiversity. Implications pertain to for whom and in what contexts specific types of dynamic emotion experiences may promote optimal functioning and resilience.

